# Targeting Neutrophil Apoptosis for Enhancing the Resolution of Inflammation

**DOI:** 10.3390/cells2020330

**Published:** 2013-05-22

**Authors:** Driss El Kebir, János G. Filep

**Affiliations:** Department of Pathology and Cell Biology, University of Montreal and Research Center, Maisonneuve-Rosemont Hospital, 5415 Boulevard de l'Assomption, Montreal, QC H1T 2M4, Canada; E-Mail: delkebirca@yahoo.fr

**Keywords:** neutrophils, apoptosis, phagocytosis, lipoxins, resolvins, annexin A1, TRAIL, cyclin-dependent kinases, Mcl-1, resolution of inflammation

## Abstract

Resolution of acute inflammation is an active process that requires inhibition of further leukocyte recruitment and removal of leukocytes from inflamed sites. Emigrated neutrophils undergo apoptosis before being removed by scavenger macrophages. Recent studies using a variety of gene knockout, transgenic and pharmacological strategies in diverse models of inflammation established neutrophil apoptosis as a critical control point in resolving inflammation. Analysis of death mechanisms revealed distinct features in executing the death program in neutrophils, which can be exploited as targets for controlling the lifespan of neutrophils. Indeed, anti-inflammatory and pro-resolution lipid mediators derived from essential fatty acids, such as lipoxin A_4_ and resolvin E1, autacoids and proteins, such as annexin A1 and TRAIL, and cyclin-dependent kinase inhibitors, can enhance the resolution of inflammation through induction of neutrophil apoptosis and promoting their removal by efferocytosis. In this review, we discuss recent advances in understanding the molecular basis of these actions, highlighting the potential of therapeutic induction of neutrophil apoptosis for dampening neutrophil-mediated tissue injury and inflammation underlying a variety of diseases.

## 1. Introduction

Neutrophils, recruited from the circulation, play a prominent role in host defense against invading pathogens. However, their many defense mechanisms, which are required for elimination of the offending micro-organisms, need to be tightly regulated to limit detrimental effects to the host [1]. Neutrophils have a short lifespan that limits expression of their pro-inflammatory functions [2,3]. During the initial phase of inflammation, neutrophils are thought to have an extended lifespan that allow appropriate expression of their defense mechanisms [1,4]. Following elimination of pathogens, emigrated neutrophils undergo apoptosis, which ensures their secure removal by scavenger macrophages through the process of efferocytosis [5,6]. Apoptotic neutrophils sequester bacterial endotoxin and cytokines [7,8] and their phagocytosis inhibits generation of pro-inflammatory cytokines [9] and polarizes macrophages into M2 (pro-resolution) phenotype [10]. These contribute to prevention of propagation of tissue damage and timely resolution of inflammation [5,11]. Delayed neutrophil apoptosis and/or impaired efferocytosis results in nonresolving inflammation, which is now considered as a critical component of many chronic human diseases, including cardiovascular diseases, diabetes and arthritis [1,5,11]. 

Ground-breaking research during the past decade has revealed that termination of inflammation is governed by active resolution programs, involving generation of a new class of lipid mediators, proteins and autacoids [11,12,13]. These endogenous molecules possess overlapping but not fully identical biological properties, including inhibition of neutrophil activation and trafficking into inflamed sites, promotion of recruitment of monocytes/macrophages and their polarization into M2 phenotype [10], enhancing neutrophil apoptosis [14,15] and facilitating efferocytosis [13,16]. This review focuses on distinct signaling pathways that govern execution of the death program in neutrophils and have been successfully targeted to induce neutrophil apoptosis to facilitate the resolution of inflammation in experimental models. 

## 2. Characteristic Features of Neutrophil Apoptosis

Mature neutrophils are terminally differentiated cells that have a short lifespan in the circulation. Neutrophil lifespan was estimated to be in the range of 8–20 h, though recent data suggest a 5.4-day lifespan in healthy humans [17]. Senescent neutrophils are thought to home to and destroyed in the spleen, liver or bone marrow [5,18]. 

During acute inflammation, extending the lifespan of neutrophils during transendothelial migration and at the sites of infection is critical for efficient destruction of invading pathogens [1,5]. Neutralization of the offending insult is generally thought to prompt emigrated neutrophils to undergo apoptosis. Apoptosis is essential for neutrophil functional shutdown. Apoptotic neutrophils sequester cytokines and endotoxin [7,8] and stimulate macrophage polarization into M2 phenotype [10], which orchestrates resolution and tissue repair [19]. Systemic injection of apoptotic neutrophils enhanced survival in animal models of sepsis [8]. In nonresolving inflammation, neutrophils persist at the inflamed site as a result of delayed apoptosis or impaired efferocytosis [1,5] and are liable to cause tissue destruction [1,20]. The dysregulated host response creates an inflammatory microenvironment with ongoing release of inflammatory mediators and damage-associated molecular patterns [1,13]. 

Altered neutrophil apoptosis is also evident under pathological conditions, though sometimes it is often difficult to decide whether prolonged survival or shortening neutrophil longevity is most favorable from the host’s perspective. For example, the opportunistic pathogen *Pseudomonas aeruginosa* [21], influenza virus A or HIV [22] accelerate neutrophil apoptosis, leading to neutropenia and compromised antimicrobial defense. In contrast, delayed neutrophil apoptosis appears to be a component of a wide range of inflammatory diseases, including acute respiratory distress syndrome (ARDS) [23], acute coronary artery disease [24], rheumatoid arthritis [25] and sepsis [26], and may be a marker of disease severity. 

The mechanisms that regulate neutrophil apoptosis have been extensively reviewed [2,3]. Neutrophils undergoing apoptosis share many similarities with other cell types, but also exhibit distinct features in executing the death program. Hallmarks of neutrophil apoptosis include pre-eminence of the Bcl-2 homologue myeloid cell leukemia-1 (Mcl-1) for maintaining neutrophil survival [27,28,29,30], restricted function of mitochondria to apoptosis [30], dependence on generation of reactive oxygen species (ROS) [30,31,32], involvement of granular enzymes in the control of apoptosis [2,3] and unusual roles for cyclin-dependent kinases [33,34,35]. These molecules also represent attractive targets to modulate life and death decisions in neutrophils.

Neutrophil survival is contingent on rescue from apoptosis by signals from the inflammatory microenvironment. Cytokines [36,37], the acute-phase reactant serum amyloid A (SAA) [38,39], and bacterial constituents [36,37,40] generate survival cues in neutrophils through activating multiple kinase pathways [41,42], ultimately leading to preservation of Mcl-1 expression and maintaining neutrophil survival. Activation of the phosphoinositide-3-kinase (PI3K) and MAPK pathways induces transcriptional activity of NF-κB, thereby generating additional survival cues [43]. Of note, survival signals, such as GM-CSF, also stimulate ROS production. However, a more robust ERK posphorylation generates a strong competing survival cue that shifts the life-death balance towards survival [44]. Studies on p38 MAPK yielded contradictory results; p38 MAPK has been implicated in inactivation of caspase-3 and caspase-8, leading to prolonged survival [45]. On the other hand, p38 MAPK-dependent reduction of Mcl-1 expression, resulting in apoptosis has also been reported [46].

## 3. Therapeutic Induction of Neutrophil Apoptosis for Enhancing Resolution of Inflammation

### 3.1. Modulation of Neutrophil Apoptosis by Outside-In Signaling through Mac-1

Mac-1, a member of the β_2_ integrin (αβ) family is expressed on circulating leukocytes [47] and best known for mediating leukocyte adhesion to the endothelium [48,49] and phagocytosis of complement-opsonized targets [50]. Engagement of Mac-1 with its ligands, ICAM-1 and fibrinogen, or opsonized bacteria generates outside-in signals to modulate neutrophil survival in a ligand and context-dependent fashion. 

Transendothelial migration of neutrophils prolongs their lifespan by delaying apoptosis through activation of the PI3K/Akt, MAPK/ERK and NF-κB survival pathways [4,51,52]. Another Mac-1 ligand is myeloperoxidase (MPO) [53,54], the most abundant enzyme stored in the primary granules in neutrophils, which is rapidly released upon neutrophil activation. MPO and MPO-generated reactive oxidants have been implicated in killing of microbes [55,56], formation of extracellular traps (NET) [57,58] as well as in inflicting tissue damage [55,59]. MPO delays constitutive neutrophil apoptosis through ERK 1/2 and PI3K/Akt-mediated preservation of Mcl-1, and prevention of mitochondrial dysfunction and activation of caspase-3 [60]. MPO binding to Mac-1 evokes superoxide generation by NADPH oxidase [54], induces release of elastase and MPO from the azurophilic granules, and up-regulates surface expression of Mac-1 [54,60], implying an autocrine/paracrine circuit for amplifying neutrophil responses to MPO [61]. MPO also delays apoptosis in emigrated neutrophils and delays spontaneous resolution of lung inflammation in a mouse model of acute respiratory distress syndrome [60]. MPO-deficiency protects mice against *Escherichia coli-*evoked lung injury [62] and ischemia-reperfusion-induced renal dysfunction and neutrophil accumulation [63], although these studies did not address apoptosis in emigrated neutrophils. 

Mac-1 mediated phagocytosis of complement-opsonized targets, including certain bacteria (e.g., *E. coli*) and yeasts, triggers neutrophil apoptosis, also referred to as phagocytosis-induced cell death [64,65,66,67]. Phagocytosis usually evokes NADPH-dependent ROS generation [68], which contributes to killing of bacteria [56] and triggers cell death through activation of caspase-8 and caspase-3 [44,66,69]. Although phagocytosis of bacteria activates the MAPK/ERK pathway [44], ROS-activated pro-apoptosis signals can effectively override such survival cues. Neutrophils from patients with chronic granulomatous disease show reduced apoptosis following phagocytosis [64]. Intriguingly, bacteria, such as *Chlamydia pneumonia* and *Neisseria gonorrheae* that survive within neutrophils following phagocytosis inhibit apoptosis [65]. 

#### 3.1.1. Lipoxins Inhibit Myeloperoxidase Signaling through Mac-1

Lipoxins, the first class of lipid mediators recognized to have anti-inflammatory and proresolving actions, are generated from arachidonic acid during cell-cell interactions [12,13]. In the presence of aspirin [70] or atorvastatin [71], cyclooxygenase-2 (COX-2) produces 15R-HETE from arachidonate, which is transformed via the 5-lipoxygenase pathway to generate 15-epi-LXA_4_. LXA_4_ and 15-epi-LXA_4_, acting predominantly through the formyl-peptide receptor 2/ lipoxin receptor (FPR2/ALX), reduce neutrophil trafficking into inflamed tissues in animal and human models through down-regulation of Mac-1 [11,12,13] and direct stimulation the SOCS-2 (suppressor of cytokine synthesis) pathway [72]. Lipoxins interrupt the MPO-mediated autocrine/paracrine loop for perpetuation of neutrophil activation, override the potent MPO-generated survival signals through Mac-1 and redirect neutrophils to apoptosis *in vitro* [61]. Thus, 15-epi-LXA_4_ attenuates activation of ERK, PI3K and NF-κB, facilitates Mcl-1 degradation, leading to collapse of mitochondrial transmembrane potential and caspase-3-mediated neutrophil death [61,73]. Treatment of mice with15-epi-LXA_4_ at the peak of inflammation enhances resolution of MPO-mediated acute lung injury in mouse models and improves the survival rate [61]. 15-epi-LXA_4_ reduces neutrophil accumulation in the airways by enhancing neutrophil apoptosis, and these actions can be prevented by the pan-caspase inhibitor zVAD-fmk [61], highlighting the importance of neutrophil apoptosis in inflammatory resolution. Lipoxins also facilitates recruitment of monocytes/macrophages, stimulate phagocytosis of apoptotic neutrophils [16,61,74] and the production of the anti-inflammatory cytokines, such as IL-10, and promote macrophage efflux to peripheral lymph nodes [11], consistent with tissue repair [13,74]. In contrast to its action in neutrophils, LXA_4_ protects macrophages from apoptosis [75]. Consistent with these findings, aspirin and lovastatin reduce acid aspiration-induced lung inflammation, in part, through stimulation of synthesis of 15-epi-LXA_4_ [76,77]. Moreover, aspirin and sodium salicylate were reported to promote neutrophil apoptosis and enhance efferocytosis in a peritonitis model [78]. These findings suggest that formation of 15-epi-LXA_4_ could, in part, explain the multiple beneficial effects of aspirin.

#### 3.1.2. Resolvin E1 Promotes Phagocytosis-Induced Neutrophil Apoptosis

Resolvin E1 (RvE1) is synthesized from the ω-3 polyunsaturated fatty acid eicosapentaenoic acid during the resolution phase of acute inflammation [79,80]. RvE1 binds to ChemR23 and (as a partial agonists/antagonist) the leukotrine B_4_ (LTB_4_) receptor BLT1 [80,81], inhibits production of inflammatory cytokines, attenuates leukocyte recruitment [81,82], and stimulates efferocytosis *in vitro* [83]. These potent anti-inflammatory and pro-resolution actions were also demonstrated in various experimental models, including peritonitis [80], polymicrobial sepsis [84], bacterial pneumonia [85] and allergic airway inflammation [86]. Consistent with these findings, results with ChemR23-deficient mice also implied an anti-inflammatory role for ChemR23 [87]. Recent data indicates that RvE1 also modulates neutrophil apoptosis [88]. RvE1 enhances Mac-1-mediated phagocytosis of complement-opsonized microbes, leading to increased ROS generation by NADPH oxidase and subsequent activation of caspase-8 and caspase-3 [88]. RvE1 also attenuates ERK and Akt-mediated survival cues generated by MPO, SAA and bacterial DNA, culminating in reduced Mcl-1 levels, thereby reinforcing the shift toward apoptosis [88]. These actions of RvE1 are predominantly mediated via BLT1 *in vitro*, indicating that resolution mechanisms may also be activated via this type of LTB_4_ receptor. RvE1 through ChemR23 stimulates phagocytosis of apoptotic neutrophils by macrophages, resulting in a macrophage phenotype switch without evoking apoptosis [80,81,83,84]. Thus, RvE1 may exert different proresolution actions via distinct receptors, and concurrent activation of these circuits may be critical for optimal resolution. These actions of RvE1 were also evident in experimental models of ARDS and bacterial pneumonia [88]. RvE1 administered at the peak of inflammation, promoted apoptosis in neutrophils emigrated into the airways, enhanced recruitment of monocytes to the airways, and facilitated clearance of apoptotic neutrophils and tissue repair [88], consistent with the original properties defining RvE1 actions [12]. Pharmacological caspase blockade prevented RvE1-induced neutrophil apoptosis and reductions in further neutrophil accumulation [88], and aggravated lung injury likely due to persisting presence of neutrophils. Eicosapentaenoic acid is also a substrate for acetylated COX-2, which generates aspirin-triggered resolvins that shares anti-inflammatory actions of native resolvins [11]. It remains to be investigated whether phagocytosis-induced neutrophil apoptosis by aspirin-triggered resolvins could contribute to the anti-inflammatory actions of aspirin. 

### 3.2. Annexin A1-Mediated Neutrophil Apoptosis

Annexin A1 (AnxA1), a member of the annexin super-family of Ca^2+^ and phospholipid-binding proteins, was originally identified as a glucocorticoid-inducible protein, which inhibited phospholipase A2 activity and hence prostaglandin generation (reviewed in [89]). Extensive studies using a combination of pharmacological and genetic approaches documented an important role for AnxA1 to inhibit inflammatory mediator production, to control leukocyte recruitment to inflamed tissues and to promote tissue repair [89,90]. AnxA1 is thought to mediate many anti-inflammatory actions of glucocorticoids [91,92]. In neutrophils, AnxA1 is rapidly mobilized from the cytoplasm to the cell surface following adherence to the endothelium [93]. AnxA1 binds to and activates FPR2/ALX and induces detachment of adhered neutrophils [94]. Emigrated neutrophils and even apoptotic neutrophils were found to release AnxA1 in a glucocorticoid-independent manner [95]. Exogenously administered AnxA1 or AnxA1 present in the inflammatory exudates induces neutrophil apoptosis through activation of caspase-3 and inhibition of Mcl-1, ERK 1/2 and NF-κB-mediated survival signals [96,97,98]. This is in sharp contrast to the apoptosis suppressing action of glucocorticoids [99]. These apparently contradictory observations might reflect differences in the functions of intracellular and extracellular AnxA1. Since glucocorticoids can augment AnxA1 contents in neutrophils [100], it is possible that AnxA1 might counteract the anti-apoptotic action of glucocorticoids at the resolution phase of inflammation. Indeed, externalization of AnxA1 on to the plasma membrane of (early) apoptotic cells may function as an “eat me” signal [101], though this has recently been questioned. Membrane-bound and exudates AnxA1 could promote phagocytosis of apoptotic neutrophils by macrophages *in vitro* [102,103], and in the normal bone marrow [104] and inflamed lung [97]. Thus, secreted AnxA1 modulates both neutrophil apoptosis and efferocytosis, which are crucial for natural as well as glucocorticoid-induced resolution of inflammation [97,98]. Two further comments about AnxA1 are in order. The first is to recall that peptide Ac2-26, an AnxA1 N-terminal-derived peptide, mimics the anti-inflammatory and proresolution actions of the full-length protein, including induction of neutrophil apoptosis in the pleural cavity [97,102]. Likewise, promising results were obtained with a cleavage-resistant mutant AnxA1 to control inflammation in the microvasculature [105], though the impact of this mutant on neutrophil apoptosis has not yet been reported. The second is a reminder that the pleiotropic receptor FPR2/ALX integrates opposing signals to determine the fate of neutrophils [106]. For example, another FPR2/ALX ligand, SAA generates potent anti-apoptosis signals that can be overridden by LXA_4_ [38], whereas excessive production of SAA was found to mediate exacerbation of glucocorticoid-refractory lung inflammation in patients with chronic obstructive pulmonary disease by overwhelming LXA_4_-generated anti-inflammatory signaling [107]. It would be interesting to know whether AnxA1 could override the actions of SAA or whether AnxA1 could act in concert with LXA_4_ to redirect neutrophils to apoptosis.

### 3.3. The Death Receptor Ligand TRAIL: A Physiological Brake to Restrain Inflammation?

Tumor necrosis factor-related apoptosis-inducing ligand (TRAIL) along with other members of the tumor necrosis factor (TNF) superfamily TNF and Fas ligand binds to death receptors and trigger the extrinsic pathway of apoptosis in many cell types [108]. Neutrophils express the TRAIL receptors TRAIL-R1 [109] and TRAIL-R2 [110]. Cross-linking these receptors recruits adaptor proteins that contain cytoplasmic death domains, leading to activation of caspase-8 and apoptosis [111]. Neutrophils produce TRAIL in response to interferon [112,113], though the biological implications of these observations had been in question. TRAIL does not appear to play a role in constitutive neutrophil apoptosis [114], whereas it has been implicated in the bone marrow clearance of senescent neutrophils [115]. TRAIL or TRAIL-R-deficient mice display increased susceptibility to acute and chronic inflammation [116], suggesting loss of TRAIL-associated proresolution mechanisms. Indeed, zymosan-induced peritonitis and LPS-induced lung injury were associated with increased neutrophil numbers concomitant with decreased neutrophil apoptosis in TRAIL-deficient mice [114]. Treatment with recombinant TRAIL 24 h after the inflammatory stimuli resulted in increased neutrophil apoptosis parallel with accelerated resolution of inflammation in both wild type and TRAIL-deficient mice [114]. Recombinant TRAIL did not appear to reduce macrophage numbers, presumably allowing efficient efferocytosis of increased apoptotic neutrophil burden. In a mouse model of acute respiratory distress syndrome (ARDS), TLR4 signaling was found to promote neutrophil apoptosis and to attenuate pulmonary inflammation through IFN-β-mediated upregulation of TRAIL [117]. These observations led to the attractive hypothesis that TRAIL may function as a physiological brake, acting only under inflammatory conditions to limit the extent of the inflammatory response [118]. However, additional studies are required to define the precise role of TRAIL, for TRAIL-deficiency had detrimental consequences in murine models of bacterial meningitis and influenza infection [116]. 

### 3.4. Cyclin-Dependent Kinase Inhibitors

One of the peculiarities of neutrophil apoptosis is an unusual role of cyclin-dependent kinases (CDKs). Freshly isolated neutrophils express the cell cycle-independent CDKs, CDK5, CDK7 and CDK 9 at the protein level [34,35], whereas contradictory findings were reported for expression of cell cycle-dependent CDKs [33,34]. Recent studies identified CDK9 as a regulator of spontaneous apoptosis in human neutrophils. Thus, CDK9 activity decreased in senescent neutrophils and correlated with decreased expression of Mcl-1 [34]. The broad range CDK inhibitor R-roscovitine as well as selective CDK9 or CDK7/CDK9 inhibitors accelerated neutrophil apoptosis, coinciding with Mcl-1 down-regulation [34,35]. The mechanisms by which CDKs regulate Mcl-1 expression remains to be elucidated, although a role for activation of RNA polymerase II and transcription of Mcl-1 gene has been proposed [34,35]. Consistently, treatment with R-roscovitine accelerated resolution in three models of inflammation (carrageenan-induced pleurisy, bleomycin-induced acute lung injury and passively induced arthritis) and this was attributed to induction of inflammatory cell apoptosis [33]. Of note, R-roscovitine also augmented apoptosis in macrophages thus might have negatively affected efferocytosis. Importantly, local delivery (*i.e.*, intra-tracheal instillation) of R-roscovitine and another CDK7/9 inhibitor DRB (5,6-dichloro-1beta-D-ribofuranosyl benzimidazole) accelerated resolution of bleomycin-induced lung injury in mice [35]. Furthermore, the CDK9 inhibitor flavopiridol was reported to effectively reduce joint inflammation in a model of rheumatoid arthritis [119], although it remains to be investigated whether this was due to acceleration of neutrophil apoptosis within the joints. The findings that *ex vivo* treatment of neutrophils from cystic fibrosis patients restored suppressed apoptosis to normal levels [120] suggest the therapeutic potential for CDK inhibitors in the clinical setting. 

### 3.5. NF-κB Inhibitors

The role of NF-κB in inflammation and the potential anti-inflammatory actions of NF-κB blockers have extensively been investigated [121], though NF-κB has also been implicated in the regulation of the resolution of inflammation [122]. Since NF-κB activation generates survival cues in neutrophils [43], inhibition of NF-κB signaling can be anticipated to redirect neutrophils to apoptosis. However, no clear picture has emerged from use of NF-κB inhibitors. For example, injection of an oligonucleotide decoy to NF-κB enhanced neutrophil apoptosis and efferocytosis in a rat model of chronic inflammation (carrageenan-sponge implant model) [123]. Increased apoptosis correlated with increases in the Bax/Bcl2 protein expression ratio. In contrast, NF-κB inhibition failed to resolve neutrophil accumulation in LPS-induced pleurisy model [124] and even prolonged inflammation and prevented neutrophil apoptosis in a carrageenan-induced pleurisy model [122]. Other studies raised concerns about the therapeutic effectiveness of NF-κB inhibition in GM-CSF-mediated pathologies, since this cytokine does not signal through NF-κB [78]. Adding to the complexity is that the route of administration of NF-κB inhibitors might determine their effectiveness. Indeed, systemic administration of a cell-permeable form of IκBα (Tat-srIκBα chimera) reduced leukocyte recruitment and enhanced caspase-3-mediated apoptosis in emigrated cells in a rat model of pleurisy, whereas local administration of Tat-srIκBα produced only marginal reductions in neutrophil accumulation [125]. These findings raise the possibility that NF-κB inhibition results in different actions from circulating and emigrated neutrophils. 

## 4. Conclusions

**Figure 1:**
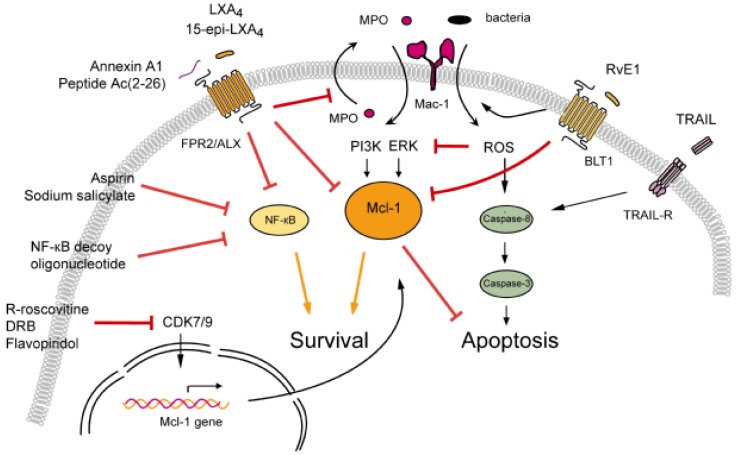
Proposed molecular mechanisms for neutrophil apoptosis-inducing agents with demonstrated pro-resolution properties *in vivo*. The adhesion receptor Mac-1 modulates the fate of neutrophils by integrating survival and pro-apoptosis cues. Ligation of the FPR2/ALX by lipoxin A_4_ (LXA_4_)/aspirin-triggered 15-epi-LXA_4_ or annexin A1/peptide Ac (2-26) counters Mcl-1 and NF-κB-mediated survival signals and redirects neutrophils to apoptosis. Lipoxins also interrupts MPO-mediated self-amplifying loop. CDK inhibitor drugs induce apoptosis via down-regulating the transcription of the key survival protein Mcl-1. RvE1 through BLT1 enhances phagocytosis of bacteria and phagocytosis-induced ROS-mediated apoptosis. TRAIL induces apoptosis through activation of caspase-8. MPO, myeloperoxidase; ROS, reactive oxygen species.

A growing body of evidence indicates that in addition to inhibiting leukocyte trafficking and facilitating neutrophil efferocytosis, anti-inflammatory and proresolving lipid mediators, such as LXA_4_ and RvE1, the anti-inflammatory protein annexin A1 and its peptidomimetics, TRAIL and cyclin-dependent kinase inhibitors can also enhance apoptosis in emigrated neutrophils (Figure 1), an important control point of the inflammatory response. Although these agents share many beneficial actions, they activate distinct molecular circuits that shift the balance of competing pro-survival and pro-apoptosis signals toward apoptosis in neutrophils *in vitro* as well as in a variety of experimental models of inflammation (Table 1). In most models, increased neutrophil apoptosis was associated with dramatic reductions in tissue neutrophil accumulation and enhanced efferocytosis, parallel with accelerated resolution of inflammation, improved clinical scores or survival rate. While clinical trials with these compounds remain distant, these results reinforce the concept of therapeutic induction of neutrophil apoptosis for limiting tissue damage and enhancing the resolution of neutrophil-mediated inflammatory pathologies. 

**Table 1:** Summary of therapeutic strategies to induce neutrophils apoptosis for enhancing the resolution of inflammation in experimental models of inflammation (ALI, acute lung injury; TRAIL, TNF-related apoptosis-inducing ligand; DRB, 5,6-dichloro-1beta-D-ribofuranosyl benzimidazole; PDE4, phosphodiesterase 4; n.d., not determined).

Disease model	Species	Compound	Effects	Pathway	Refs
Carrageenan-induced pleurisy	Mouse	R-roscovitine	Enhanced PMN apoptosis and efferocytosis Reduced lung PMNs and monocytes	n.d.	[33]
	Rat	IkBα repressor	Enhanced leukocyte apoptosis Reduced tissue inflammatory cells	Increased caspases-3 activity	[125]
Carrageenan plus MPO-induced ALI	Mouse	15-epi-LXA_4_	Enhanced PMN apoptosis and efferocytosis Decreased PMN accumulation Increased lung monocytes/macrophages	Reduced Mcl-1, ERK and PI3K	[61]
	Mouse	Resolvin E1	Enhanced PMN apoptosis and efferocytosis Decreased PMN accumulation Increased lung monocytes/macrophages	Reduced Mcl-1 Enhanced phagocytosis	[88]
*E. coli* peritonitis-associated ALI	Mouse	15-epi-LXA_4_	Enhanced PMN apoptosis and efferocytosis Decreased PMN accumulation	Reduced Mcl-1, ERK and PI3K	[61]
		Resolvin E1	Enhanced PMN apoptosis and efferocytosis Decreased PMN accumulation	Reduced Mcl-1 Enhanced phagocytosis	[88]
*E. coli*-induced pneumonia	Mouse	Resolvin E1	Enhanced PMN apoptosis and efferocytosis Decreased PMN accumulationIncreased lung monocytes/macrophages	Reduced Mcl-1 expression	[88]
LPS-induced ALI	Mouse	MetforminRotenone	Decreased PMN accumulation	Decreased NF-κB activation	[126]
	Mouse	Nutlin-3a	Enhanced PMN apoptosis	Increased p53 expression	[127]
	Mouse	rTRAIL	Enhanced PMN apoptosisReduced PMN accumulation No effect on macrophage number	Activation of caspases-8	[114]
LPS-induced pleurisy	Mouse	Rolipram	Enhanced PMN apoptosis Reduced lung PMNs	Enhanced PDE4 activityReduced PI3K/Akt	[124]
	Mouse	Annexin A1 andpeptide Ac(2-26)	Enhanced PMN apoptosis Reduced PMN accumulation	Reduced Mcl-1, ERK and NF-κB	[97]
	Mouse	Cleavage-resistant annexin A1	Reduced PMN accumulation	n.d.	[105]
Bleomycin-induced lung injury	Mouse	R-roscovitine	Enhanced PMN apoptosis	Decreased Mcl-1	[33]
	Mouse	CDK7/9 inhibitor DRB	Enhanced PMN apoptosis	Decreased Mcl-1 transcription	[35]
Collagen-induced arthritis	Mouse	Flavopiridol	Reduced joint infection Cellular targets were not identified	n.d.	[119]
Passive arthritis	Mouse	R-roscovitine	Improved clinical scores	n.d.	[33]
Thyoglycollate-induced peritonitis	Mouse	Aspirin Sodium salicylate	Enhanced PMN apoptosis and efferocytosis	Inhibition of NF-κB	[78]
Zymosan-induced peritonitis	Mouse	rTRAIL	Enhanced PMN apoptosis Reduced PMN accumulation No effect on macrophage number	Activation of caspases-8	[114]
Pneumococcal meningitis	Mouse	R-roscovitine	Enhanced PMN apoptosis Alleviated brain damage	Reduced Bcl-2 expression	[128]
Subcutaneous sponge-implant	Rat	NF-κB decoy oligonucleotide	Enhanced PMN apoptosis and efferocytosis	Increased Bax, reduced Bcl2	[123]
